# Supplementary material for: Knowledge, attitudes, and perceptions of healthcare students and professionals on the use of artificial intelligence in healthcare in Pakistan
Source: PLOS Digit Health. 2024 May 10;3(5):e0000443. doi: 10.1371/journal.pdig.0000443 (PMC11086889; doi:10.1371/journal.pdig.0000443)
Supplement: S1 Questionnaire — (PDF) [file pdig.0000443.s001.pdf]

Knowledge, attitudes, and perceptions of healthcare professionals on the use of artificial intelligence in healthcare – A cross-sectional study

Thank you for being willing to participate in this study! The data you will provide is very valuable and will be used to gauge the knowledge, attitudes and perceptions of healthcare professionals i.e doctors, nurses, medical students and allied health professionals. This will help guide the necessity and the future of AI in healthcare.

Attached below is the consent form. After reading it, if you decide to participate in, click the "Yes, I consent" button at the end of this section.

* Indicates required question

1. ADULT RESEARCH SUBJECT - INFORMED CONSENT FORM *

Title: Knowledge, attitudes, and practices of healthcare professionals on the use of artificial intelligence in healthcare.

Investigators - Dr. Rehman Siddiqui, Dr. Zahra Hoodbhoy, Muhammad Mustafa Habib

Purpose: You are invited to participate in the research project entitled "Knowledge, attitudes, and practices of healthcare professionals on the use of artificial intelligence in healthcare - a cross sectional study ". By participating in this survey, you will be helping provide information that may be essential in driving research and education about AI in medicine.

Background - Artificial Intelligence (AI) has been rapidly developing in recent years and is starting to transform various fields, including medicine. AI has the potential to revolutionise healthcare by improving diagnosis, treatment, and patient outcomes. A survey aimed at assessing their knowledge and attitudes about AI in medicine will help identify the gaps in understanding and inform strategies to educate and train healthcare professionals and students.

Procedure - This research survey will take place digitally. This form will be distributed via social media e.g., Facebook, WhatsApp etc. and email. The prompts are divided into 5 sections and complete form is estimated to take 4-5 minutes to complete.

The survey is completely anonymous with no protected health information (PHIs) nor personal identifiers which reduces the risks of losing confidentiality or anonymity. The data will be researched and analysed by only the principal investigator and the members of the research team.

For further questions at any time before, during or after your participation in this study you may contact a member of the research team.

You are making a decision whether or not to participate in this research study. Clicking "Yes, I agree" and completing the survey will act as your agreement.

By participating in this research, you confirm that you are at least 18 years old.

Mark only one oval.

- I agree
- I disagree

Section 1 - Demographical data

2. What is your age in years? *

3. What is your gender? *

Check all that apply.

- Male
- Female
- Prefer not to say

4. What is your highest educational qualification? (If the program you are enrolled in is your highest educational qualification, please select that below e.g students in medical school will select the option labeled " bachelor degree ") *

Mark only one oval.

- Below matriculation level or equivalent
- Secondary school Certificate (SCC) - Matric
- Higher Secondary School Certificate (HSSC)
- Associate Ordinary Bachelor
- Bachelor Degree
- Masters Degree
- Doctoral Degree

5. Are you studying/working in a government or a private healthcare institute or set-up?

Mark only one oval.

- Private
- Government
- Other:

6. What is the name of your healthcare institute/ university?

7. What is your source of information about artificial intelligence? *

Check all that apply.

- Media
- Social media
- Web Browsing
- Friends/Family
- University/workplace
- Scientific journals
- Other:

8. What is your current role in your respective healthcare setup? *

Mark only one oval.

- Undergraduate Student
- Doctor
- Nurse
- Allied Health professional
- Other

9. What program are you currently enrolled in? *

Mark only one oval.

- I am not a student
- MBBS (Bachelor of Medicine and Bachelor of Surgery)
- BScN (Bachelor of Science in Nursing)
- ASDH (Associate of Science in Dental Hygiene)
- PGME (Post Graduate Medical Program)
- Other:

10. What year of study are you in? *

Mark only one oval.

- I am not a student
- 1st Year
- 2nd Year
- 3rd Year
- 4th Year
- 5th Year
- Other:

11. Are you working in a clinical role currently?e.g rotating through clinical rotations. *

Mark only one oval.

- I am not a student
- Yes
- No

12. When should training in artificial intelligence competencies begin to better prepare students for clinical practice? *

Mark only one oval.

- At the undergraduate level
- During residency/ Internship
- After becoming a practicing healthcare provider
- No training is necessary

Section 3 - Knowledge of AI

13. Have you ever had any formal sessions in artificial intelligence in your studies/employment? *

Mark only one oval.

- Yes
- No

14. Do you consider yourself technologically adept? i.e proficient in the use of modern technology? *

Mark only one oval.

- Strongly disagree
- Somewhat disagree
- Neutral
- Somewhat agree
- Strongly agree

15. Answer the prompts listed below. *

Mark only one oval per row.

	Yes	Maybe	No
**Artificial intelligence is an umbrella term encompassing many technologies (e.g., machine learning, deep neural networks etc.) Are you aware of these terms?**			
**Many applications we use in daily life already use AI (e.g speech/text recognition, email spam filters). Are you familiar with these applications?**			
**Are you familiar with AI Applications in healthcare e.g robotic surgery?**			

16. What may be useful ways for you to explore and learn about AI? (Select all that apply) *

Check all that apply.

- Q&A panels with experts
- AI Symposiums with experts and their AI-related research
- Student led- journal clubs
- Formal preclinical elective program
- Workshops on programming AI models
- Access to programming workshops (i.e python, R etc.) and various online resources
- Other:

Section 4 - Perceptions about AI

17. Do you believe that AI can perform the tasks mentioned below at a level comparable to that of a human healthcare professional? *

Mark only one oval per row.

	Extremely unlikely	Unlikely	Neutral	Likely	Extremely likely
**Provide patients with preventative health recommendations (e.g. exercise, diet, wellness).**					
**Analyze patient information to reach diagnoses.**					
**Analyze patient information to establish prognoses.**					
**Read and interpret diagnostic imaging.**					
**Evaluate when to refer patients to other health professionals.**					
**Formulate personalized treatment plans for patients.**					
**Formulate personalized medication prescriptions for patients.**					
**Provide empathetic care to patients.**					
**Monitor patient compliance to prescribed medications, exercise and dietary recommendations.**					
**Provide psychiatric/personal counselling.**					
**Perform surgery (e.g. robotic surgery).**					
**Provide documentation (e.g., update medical records) about patients.**					
**Assist hospitals in capacity planning and human resource management.**					
**Provide recommendations for quality improvement in practices/hospitals.**					
**Conduct population health surveillance and outbreak prevention.**					

Section 5 - Attitudes about AI

18. In your opinion, will artificial intelligence impact the medical profession? Answer the prompts mentioned below. *

Mark only one oval per row.

	Strongly disagree	Somewhat disagree	Neutral	Somewhat agree	Strongly Agree
**Artificial Intelligence will reduce the number of jobs available to physicians**					
**Artificial Intelligence will reduce the number of jobs in certain medical specialties more than others.**					
**Artificial Intelligence has impacted my choice of specialty selection.**					
**AI in medicine will raise new ethical challenges.**					
**AI in medicine will raise new social challenges.**					
**AI in medicine will raise new challenges around health equity.**					
**The Pakistani healthcare system is currently well prepared to deal with challenges having to do with AI.**					
**My medical education is adequately preparing me for working alongside AI models.**					
**Medical training should include training on AI competencies.**					
**Every medical trainee should be required to receive training in AI competencies.**					

19. Answer the questions below. *

Mark only one oval per row.

	Strongly disagree	Somewhat disagree	Neutral	Somewhat agree	Strongly Agree
**Artificial intelligence will improve healthcare in general.**					
**The non-interventional physician will be replaced in the foreseeable future.**					
**In the foreseeable future, all physicians will be replaced.**					
**These developments frighten me.**					
**These developments make healthcare in general more exciting to me.**					
**Artificial intelligence will never make the human physician expendable.**					
**Artificial intelligence should be part of a healthcare professional's training.**					

20. How much time a month would you like to learn about artificial intelligence? *

Mark only one oval.

- I am not interested in learning about artificial intelligence
- <30 minutes
- 1 hour
- 1-5 hours
- 5-10 Hours
- >10 Hours

21. Please use this space to share any other thoughts on the topic that you feel are significant but weren't completely captured in the survey.

This content is neither created nor endorsed by Google.

Google Forms
